# A rare case of ovarian adenomyoma mimicking primary invasive ovarian cancer with a contralateral serous borderline ovarian tumor: A case report and review of the literature

**DOI:** 10.1016/j.heliyon.2020.e04406

**Published:** 2020-07-24

**Authors:** Viola Liberale, Alessandra Surace, Lorenzo Daniele, Luca Liban Mariani

**Affiliations:** a Academic Department of Gynecology and Obstetrics, University of Turin, Mauriziano Hospital, Turin, Italy; b Academic Department of Gynecology and Obstetrics 2, Department of Surgical Sciences, City of Health and Science, University of Turin, Turin, Italy; c Department of Radiology, Mauriziano Hospital, Turin, Italy

**Keywords:** Abdominal surgery, Cancer surgery, Gynecology, Medical imaging, Radiology, Extrauterine adenomyoma, Ovarian adenomyoma, Uterus-like mass, Borderline ovarian tumor, Ovarian cancer

## Abstract

Extrauterine adenomyoma is a rare type of benign tumor, characterized by nodular aggregate of smooth muscle, endometrial glands and endometrial stroma, arising outside the uterus. In this study we describe a case of primary ovarian adenomyoma associated with endometriotic cysts with contralateral serous borderline tumor in a 40-year-old woman and we highlight how preoperative exams could lead to the suspicious of invasive cancer. We provide a review of the literature, analyzing all cases of extrauterine adenomyoma published so far, classifying them on the basis of pathogenetic theories proposed, localization of the lesion, imaging modalities and treatment adopted.

## 1 Introduction

An extrauterine adenomyoma is a circumscribed, nodular aggregate of smooth muscle, endometrial glands and endometrial stroma originating outside the uterus. This rare type of benign tumor has been described in pararectal spaces, ovaries, broad ligament, peritoneum, cornus medullaris, bowel and liver [1, 2]. The ultrasound appearance is typically that of malignant ovarian tumors due to the prevalent solid component and atypical vascularization. Herein, we describe a clinical case of an ovarian adenomyoma in a symptomatic woman without a previous history of pelvic endometriosis and we provided a review of the inherent literature.

## 2 Case

A 40-year-old woman para 0000 was referred to our Institution for a pelvic pain irradiated to the left flank. Her medical history was unremarkable and she did not assume regular medications. She did not report any general surgical procedure except for a cesarean section in 2015 for breech presentation. Her family history for gynecological malignancies was negative. At admission, she denied dyspepsia, bowel or urinary habits changes and she had regular menses. The pelvic pain onset was intermittent and persistent over the previous three weeks.

Vital parameters were regular and the patient was apyretic. Clinical examination revealed a left iliac firm and painful mass with tenderness exacerbated by bimanual mobilization. On palpation the uterus and right adnexa appeared physiological and no blood nor atypical vaginal discharge was recorded. Blood tests were all in normal ranges. Alpha-fetoprotein, carcinoembryonic antigen, CA-19.9, CA-15.3, were all negative. Serum cancer antigen-125 was elevated, reaching 680.8 UI/ml.

**Figure 1:**
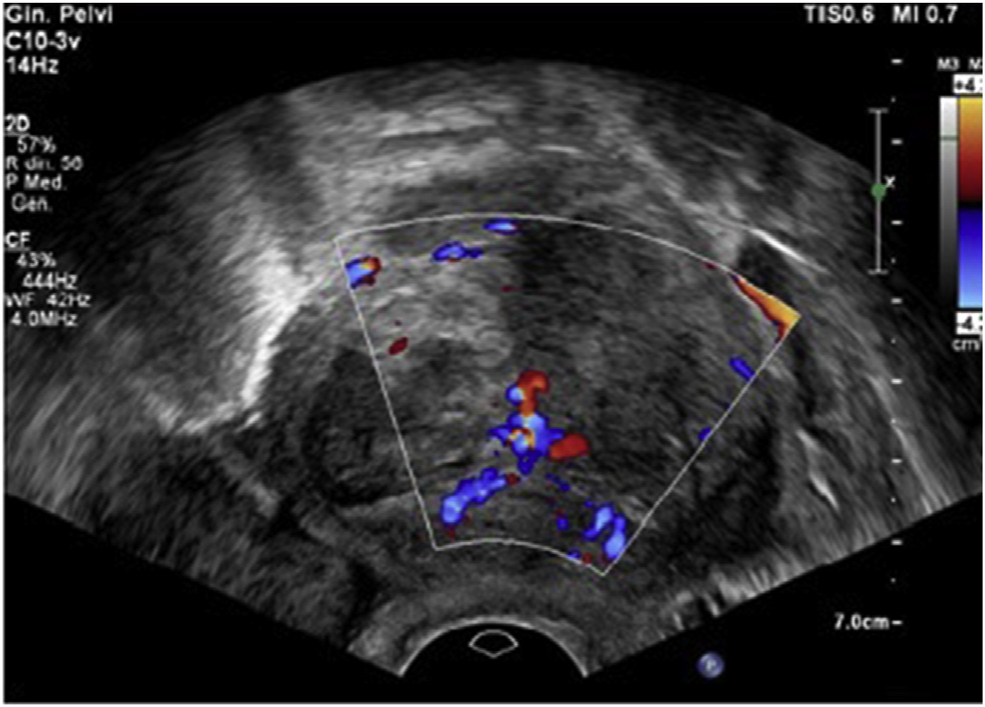
Left ovary ultrasound imaging: irregular dishomogeneous solid mass of 63 × 62 × 60 mm with two hypoechoic cysts and regular margins. Color score 4.

Transvaginal scan was performed by using a 5–7 MHz transvaginal transducers (Affiniti 70 - Philips). IOTA (International Ovarian Tumor Analysis) terms and definitions were adopted to describe the ovarian lesion. The ultrasound (US) examination confirmed the presence of an irregular dishomogeneous solid mass of 63 × 62 × 60 mm, arising from the left ovary, with two hypoechoic cysts and regular margins (Figure 1).

**Figure 2:**
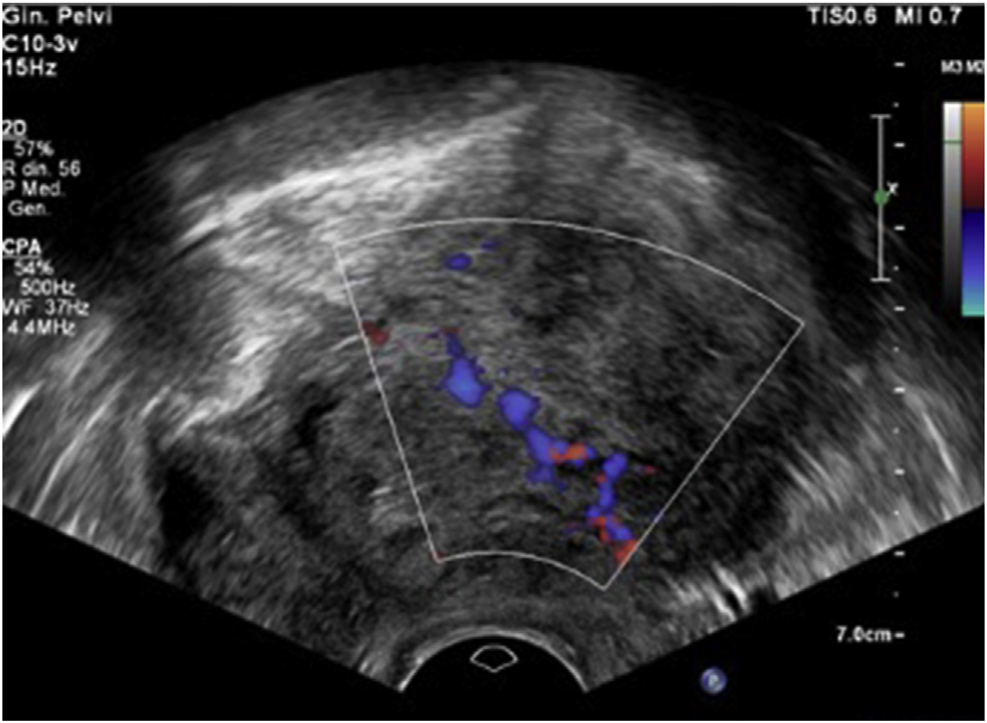
A single dominant vessel crossing the central part of the left ovary mass.

The operator attributed a color score of 4 (highly vascularized) due to the presence of a single dominant vessel crossing the central part of the mass with multiple branches distributing to the periphery and surrounding the cystic areas (Figure 2, video 1).

Supplementary data related to this article can be found online at https://doi.org/10.1016/j.heliyon.2020.e04406

The following are the Supplementary data related to this article:

Video 1 Left ovary lesion, suspected for invasive epithelial ovarian cancer. Video 1

Video 2 Right ovarian lesion suspected for borderline ovarian tumor. Video 2

**Figure 3:**
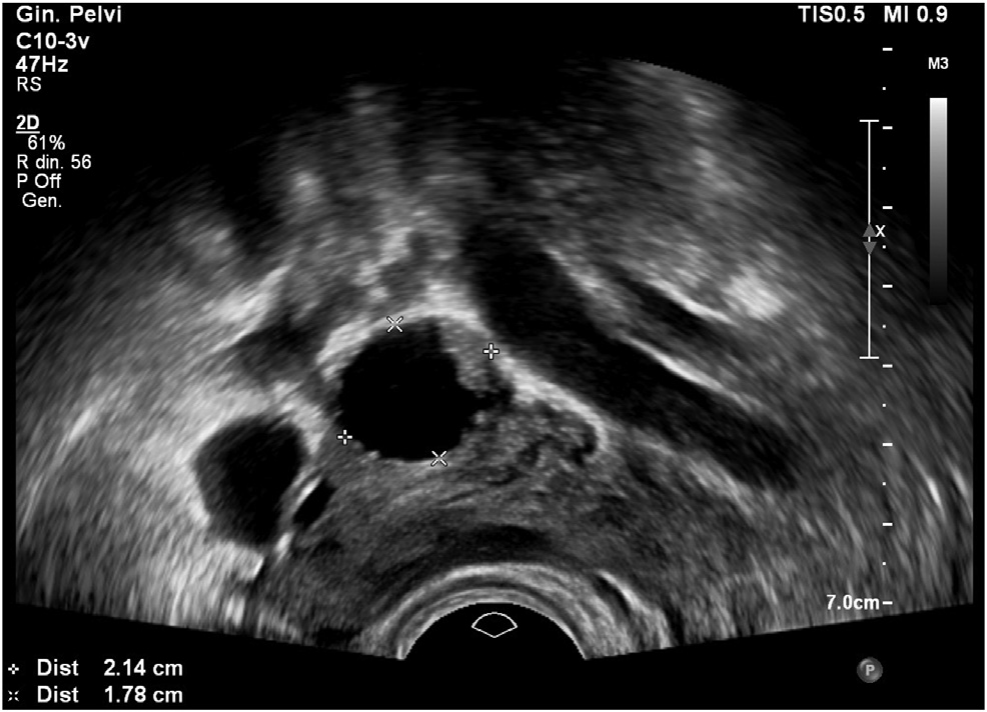
The unilocular cystic right ovarian lesion with irregular borders suspected for ovarian borderline tumor.

**Figure 4:**
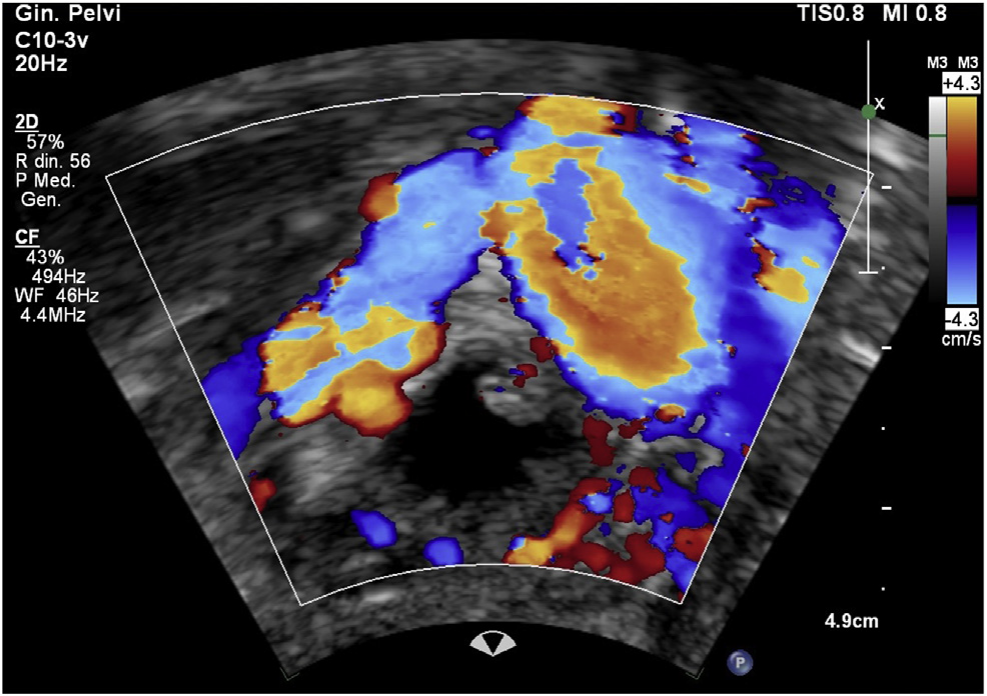
Color score 3 of the right ovarian lesion suspected for borderline ovarian tumor.

**Figure 5:**
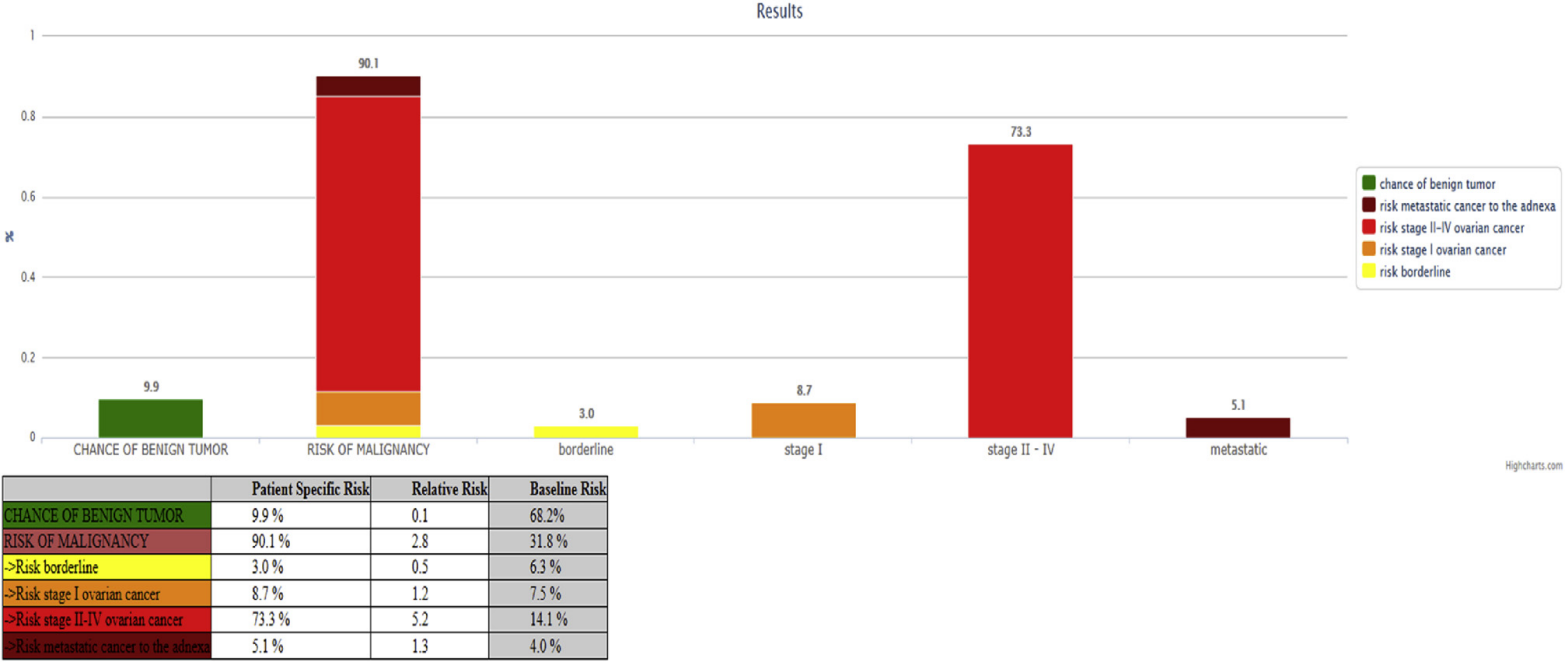
ADNEX model of left ovary mass.

A positive sliding sign was present between the uterus and the pelvic sidewall. Furthermore, the right adnexa appeared to have an increased volume (55 × 31 mm) with an inner hemorrhagic area and an adjacent unilocular cystic lesion with irregular borders, multiple papillary structures and color score 3 (Figures 3 and 4, video 2); the bigger papillary projection measured 11 × 11 mm. The ultrasound aspect was presumed suggestive for at least a serous borderline ovarian tumor (sBOT). The uterus had an irregular myometrial-endometrial junction with hyperechoic areas dispersed in the myometrium, a mild and diffuse ultrasound beam absorption, overall suggesting the presence of adenomyosis. No abnormalities of the bladder, ureters in the pelvic tract, or kidneys were detected. No free fluid was detected in the Douglas pouch. The overall features of the left ovarian lesion, were highly suspicious for an invasive epithelial ovarian cancer. This result was also supported by ADNEX model analysis [3] retrieving a risk of ovarian cancer of 90.1% with a risk of II - IV stage of 73.3% for the left ovary mass (Figure 5).

An overall staging was completed with thorax and a whole abdominal computed tomography (CT), which confirmed the finding of an inhomogeneous adnexal solid-cystic left mass with an irregular contour, high contrast enhancement and a smaller contralateral solid-cystic lesion. No additional lesions were reported in the remaining abdomen nor in the thorax.

An alleged diagnosis was malignant ovarian tumor, a comprehensive surgical staging was scheduled. Despite full information about her clinical condition, the patient refused concomitant hysterectomy, except in case of histological confirmation of an invasive tumor; she gave valid consent to bilateral adnexectomy and surgical abdominal staging. An endometrial biopsy was pre-operatively performed to exclude a coexisting uterine malignancy. At laparotomy, a left pelvic adnexal mass measuring 8 cm in greatest dimension was found. Furthermore, a right pelvic adnexal mass, measuring 5 cm in greatest dimension. This was adherent to sigma and uterine posterior wall; at the frozen intraoperative analysis two other oval nodes were found near the ovarian lesion (corpus luteum and endometrioma, respectively).

No additional lesions were found on exploration of pelvis and abdomen. The left adnexectomy was performed without complications and sent for frozen intraoperative analysis retrieving endometriotic cyst with hemorrhagic areas. Right adnexectomy was performed and the frozen section preliminary confirmed a tumor with borderline characteristics. Residual surgical staging, including peritoneal washing, multiple peritoneal biopsies and omentectomy, was performed. No suspicious lesions were found, lymphadenectomy was not performed because metastasis to the lymph nodes is not known to affect survival or recurrence [4].

**Figure 6:**
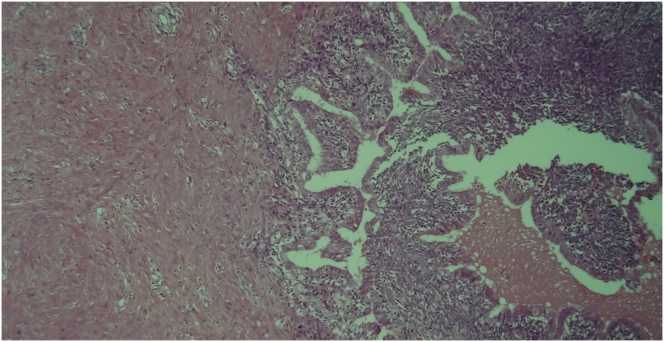
Adenomyoma with uterine-like features. Endometrial cyst cavity with blood inside is lined by typical endometrial glandular epithelium and stroma surrounded by hypertrophied smooth muscle resembling that of the myometrium.

**Figure 7:**
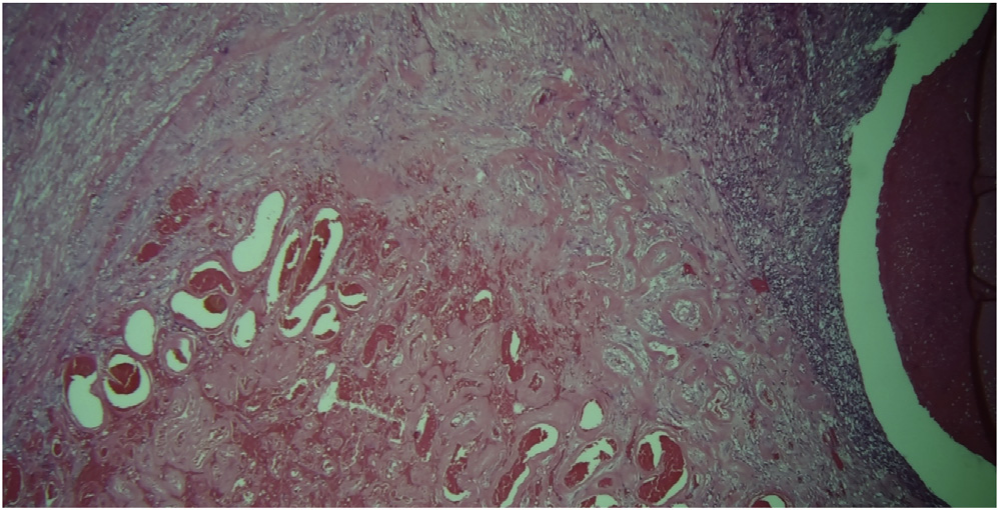
Microscopic analysis revealing the interface between hyperplastic smooth muscle layer and epithelial capsule of endometriotic cyst. The multiple vessels interspersed in the muscular layer account for high vascularization observed on ultrasound examination.

**Figure 8:**
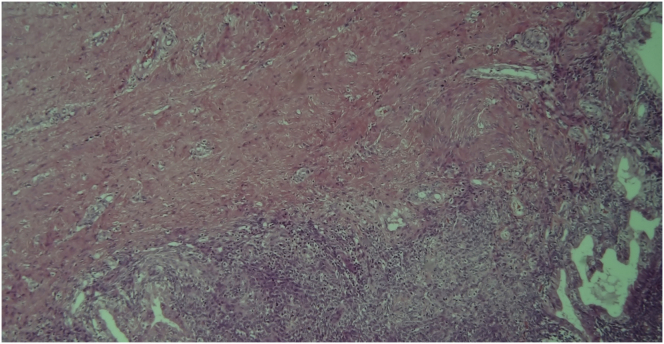
The thickened muscular layer is composed of normal, organized myometrial-type smooth muscle. The differential diagnosis is between the typical endometriomas which may also show some degree of smooth muscle metaplasia and extrauterine leiomyoma where the muscular layer is predominant with only few endometrial-type glands and stroma inside.

**Figure 9:**
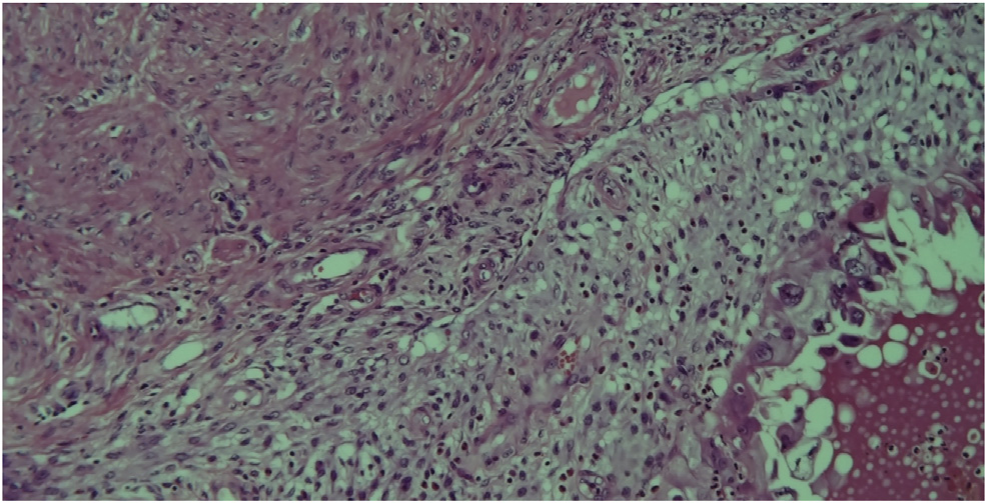
A particular of the ectopic endometrium with endometrial-type glands and stroma surrounding the cyst cavity.

Upon gross examination, the left ovary lesion was greyish, lobulated, with a smooth surface, an irregular shape and had solid-elastic consistency. On the cut section, the mass showed a prevalent solid component with gray cystic areas filled with brown-chocolate fluid. Microscopy revealed the presence of two endometriotic cysts surrounded by a variable thickness of smooth muscle layers lined with endometrial glands and stroma without nuclear atypia resembling normal uterine myometrium and endometrium. These findings were consistent with uterus-like extrauterine adenomyoma associated with ovarian endometriomas (Figures 6, 7, 8 and 9). In the same ovary a cystic-hemorrhagic corpus luteum was also found. On the cut section, right ovary mass showed a prevalent cystic component filled with clear fluid and projecting papillary structures. The diagnosis of right atypical proliferative serous tumor (according to the last WHO classification of ovarian neoplasm) was established*.* [5] All other specimens were negative for premalignant or malignant cells. The patient was discharged from hospital 4 days after surgery in good condition. Informed consent for scientific publication was obtained from the patient.

## 3 Discussion

An extrauterine adenomyoma is a rare type of benign tumor, mainly located in ovaries. Since it was first described by Cozzutto et al. in 1981 [6] subsequent cases were reported with various names as “uterus-like mass”, “extrauterine adenomyoma” and “endomyometriosis”. The pathogenesis behind extrauterine proliferation of adenomyomas is not yet well understood**.**

Cozzutto in his paper, proposed the theory in which adenomyomas could originate after a process of metaplastic transformation of endometriotic cells into smooth muscle, but this theory could not explain whole cases published later. Four other theories, from Rosai, Redman, Batt and Belmarez, have been proposed for explaining the pathophysiology of extrauterine adenomyomas and are described below.

Rosai [7] suggested the theory of defective müllerian duct fusion. This theory explains cases of extrauterine adenomyoma accompanying congenital urogenital abnormalities like renal agenesis and double excretory system associated with anomalies of the genital tract. Abnormalities of the uterus, such as rudimentary horn or uterine duplications, could lead, after a process of detachment, to an implant of a uterus-like mass in the abdominal cavity [1, 25, 52].

Since some extrauterine adenomyomas responded to hormonal treatment, in 2005 Redman et al. [25, 32, 33] suggested the theory of sub-coelomic mesenchymal metaplasia according to which multipotent cells, contained below the mesothelial layer of the peritoneum, could diﬀerentiate and grow under estrogen impulse, leading to the formation of a supernumerary müllerian uterus-like structure.

Batt et al. [8, 9] proposed the theory of mullerianosis which states that a heterotropic organoid structure of embryonic origin composed of müllerian cell rests may get incorporated into normal organs at the time of organogenesis. The müllerianosis’ theory was particularly suitable for providing an explanation for extrauterine lesions that occurred in unusual sites outside the pelvic and lower abdominal cavities. Newsworthily Belmarez et al. [54] in 2019, describing a patient with leiomyomatosis peritonealis disseminata and extrauterine adenomyomas, shed light on the possibility of a similar pathogenetic theory. Both of these pathologies could arise by deposits of iatrogenic dropped cells within the abdomen and pelvis during hysterectomy or myomectomy.

Most patients with ovarian adenomyoma had a presumptive ultrasound diagnosis of ovarian endometrioma. Moreover, most ovarian adenomyomas arise in the left ovary according to our case report. Several Authors [10, 11, 12] observed higher frequency and/or more severe pelvic endometriotic lesions on the left pelvic side due to the presence of sigma causing an anatomical distortion for the refluxing menstruation. The back-flow hypothesis may therefore, be suitably applied to ovarian adenomyoma in patients with a concomitant endometriosic lesions reinforcing Cozzutto's theory [6]. Guerriero et al. defined typical ultrasound features of ovarian endometriomas as unilocular, ground-glass cyst, with no or scarce vascularization (color score 1 and 2, respectively) [13]. More recently Van Holsbeke [14] revised the previous definition reporting that most endometriomas are premenopausal, 1–4 loculi, ground glass cysts with or without papillary projections, not vascularized. It is noteworthy that ovarian endometriomas may change their ultrasound appearance across different ages. Indeed as age increases, multilocular cysts and cysts with papillations and other solid components become more common, while the typical ground glass echogenicity of cyst fluid and tender mass on an ultrasound scan become less common [15]. These morphological changes are typically found during the fourth and fifth decades. This observation accounts for the confusion with other benign ovarian lesions or with ovarian malignancy [16]. In our case the prevalent solid component and the high and atypical vascularization (single dominant vessel with multiple branching) oriented towards a malignant lesion. Additional misleading factors were no history of pelvic endometriosis nor infertility. Moreover, ultrasound examination did not find any sign indicative or suspicious for endometriosis (i.e. uterine adenomyosis, kissing ovaries, ground glass ovarian cyst, pelvic adhesions with negative sliding signs) [17].

Notwithstanding the patient complained of pelvic pain which is a symptom often associated with endometriosis and a parameter introduced in LR1 (Logistic Regression) model of IOTA group to identify benign ovarian masses [18, 19]. The suspicious ultrasound features appear to stem from the microscopical analysis of ovarian adenomyoma as opposed to endometriomas. Indeed, several Authors depicted primary ovarian adenomyoma as a mass with central cavities lined by endometrial-type glands and stroma surrounded by well-formed and thick smooth muscles layers [20, 21]. In the present case, the final aspect of the left ovary mass was even more misleading due to the concomitant presence of endometriomas and a suspicious lesion contralaterally.

In 2018, a review of literature of extrauterine adenomyoma was published by Paul [1] and our analysis supplements Paul's review with the last published literature (Table 1). To the best of our knowledge only 42 cases of primary ovarian adenomyoma, including our case report, were published.

**Table 1:** Description of extrauterine adenomyomas. RIF- Right iliac fossa, TAH with BSO- Total abdominal hysterectomy with bilateral salpingo-oophorectomy, HRT-Hormone replacement therapy, CT-Computerized tomography, USG-Ultrasonography, IVP-Intravenous pyelography, MRI-Magnetic resonance imaging, IVU-Intravenous urography, TLH with BSO-Total laparoscopic hysterectomy with bilateral salpingo-oophorectomy, PID-Pelvic inflammatory disease, LSO-Left salpingo-oophorectomy, RSO- Right salpingo-oophorectomy, DUB- Dysfunctional uterine bleeding, GnRH- Gonadotropin releasing hormone, SCH-Supracervical hysterectomy, C – Cozzutto's theory, R- Rosai's theory, S- sub-coelomic mesenchymal metaplasia, M-mullerianosis's theory, B- Belmarez’ theory. Courtesy of Paul et al.

Sr.n	Study (Year)	Size and location	Age	Past history	Presenting complaints	Imaging modalities	Suspected pre-operative diagnosis	Surgical intervention	Pathogenic Theory
1	Rahilly et al. [23]	5 cm, right ovary	38		RIF and pelvic pain	IVP		TAH with BSO	-
2	Horie et al. [24]	14 × 11 cm, small bowel mesentery	59		lower abdomen mass	not reported		Surgical excision	M
3	Redman et al. [25]	5 cm, pararectal	50	TAH with BSO + HRT	Dysuria, suprapubic and pelvic pain	CT, USG, IVP		Excision + left ureteric stenting	B, M
4	Bayar et al. [26]	7.5 cm, left ovary	38	Gonadotropin treatment	Infertility and pelvic pain	USG		Laparoscopic excision	-
5	Choudhrie et al. [27]	0.8 cm, left ovarian ligament	57		Lump lower abdomen and pelvic pain	USG, IVU		TAH with BSO	M
6	Kim et al. [28]	10.5 × 9.5 cm, pararectal	42		Lower abdominal pain	CT		Surgical excision	M
7	Menn et al. [29]	6 × 4 cm, right broad ligament	37	Myomectomy and polypectomy	Right quadrant pain and intermenstrual spotting	USG, MRI		TAH	B
8	Kaufman et al. [30] Case 1	7 × 5 cm, right pelvic wall absent right kidney, absent right ureter	39	Subfertility, PID	Dysmenorrhea, pain and menorrhagia	USG, CT	myoma	Laparoscopic excision	R
9	Kaufman et al. Case 2	10.5 × 9 cm, right pelvic wall	57	RSO, TAH + LSO for wall endometriosis + HRT	RIF pain, suprapubic pain and backache	USG, CT,IVP	endometrioma	Laparoscopic excision+ oral medroxyprogesterone	M, C, S
10	Stewart et al. [31] Case 1	6 × 4.5 cm, left paraovarian mass	40	TAH for DUB	Left iliac fossa pain	USG		Laparoscopic excision	-
11	Stewart et al. Case 2	6.3 × 4 cm, right parametrial mass	65	PID, breast cancer	Pelvic mass	USG		Hysterectomy with BSO with mass excision	-
12	Carinelli et al. [32] Case 1	10 cm sigmoid, 6 cm pelvic, 4 cm ileal, 1 cm paraileal and paravesical	46	Myomectomy	Abdominal pain and constipation	USG,CT		Excision, hysterectomy with partial colectomy and Meckel diverticulum resection + GnRH agonist	M, B
13	Carinelli et al. Case 2	3 cm sigmoid, 3.5 cm right ovary	39	Left ovariectomy for ovary endometriosis	Dysmenorrhea, chronic abdominopelvic pain	USG,CT,MRI		Laparoscopic excision. Partial colectomy with colostomy 7 days later + GnRH agonist for relapse	M, C, B
14	Liang et al. [33]	4 cm, left broad ligament	17	Mesosalpinx cystectomy	Dysmenorrhea and pelvic pain	USG,CT		Excision	-
15	Sisodia et al [34]	5.5 × 5.3 cm, right ovarian ligament	56		Dysuria, lower abdominal pain, vaginal bleeding	USG,IVP		TAH with BSO	-
16	Moon et al. [35]	7 × 6 cm, pararectal	41	SCH and right salpingectomy		USG,MRI		Excision and LSO	M, B
17	Seki et al. [36]	3.8 × 2 cm, left inguinal region	44	Left oophorectomy, Endometriosis	Abdominal pain	USG,MRI		Surgical excision	M
18	Takeda et al. [37]	3.8 × 3.7 cm, left ovarian ligament	39		Pain lower abdomen	CT, MRI,IVP		Laparoscopic excision	-
19	Moghadamfalahi et al. [38]	6 cm, pararectal; 7.5 cm, upper abdomen	39	SCH, cervical myomectomy, endometriosis	Abdominal pain and rectal bleeding	CT		Surgical Excision	M, C
20	Carvalho et al. [39] Case 1	Few mm to 50 mm, pelvic and abdominal peritoneum and omentum, left ovary	32	Hysteroscopic myomectomy		USG, CT,MRI		Excision + Goserelin + Anastrazole	M, S
21	Carvalho et al. Case 2	Few mm to 20 mm, pelvic and abdominal peritoneum and omentum	41		Dysmenorrhea and pelvic pain, proctalgia	not reported		LSO with partial excision of nodules + Medroxy progesterone acetate	M, S
22	Kim et al. [21]	2 × 1.5 cm, appendix	46	Supracervical hysterectomy	Right lower quadrant pain	USG,CT		Surgical excision	B
23	Huanwen et al. [40]	3.6 × 2.6 cm, liver	29	Myomectomy	Back pain	USG, CT		Surgical excision	B, M
24	Bulut et al. [41]	5–10 cm, bilateral broad ligament, ectopic adrenal tissue	56		Menorrhagia and pelvic pain	USG,MRI	large necrotic leiomyoma without an exclusion of malignancy	TAH with BSO and excision of intraligamentary masses	M
25	Na et al. [42]	Caecum, descending colon and mesocolon	39	Total hysterectomy with LSO, RSO for endometriosis	Right lower quadrant pain	USG,CT	Ovarian endometriosis	Colonoscopic and laparoscopic resection	M, B, C
26	Ulm et al. [43]	3 cm, left round ligament	49		Metromenorrhagia	CT	inguinal adenopathy	TAH with BSO and lymph node dissection	M
27	Torres et al. [44]	4 cm, right broad ligament	58		Post menopausal bleeding	USG,CT	malignancy	Total Robotic hysterectomy with bilateral salpingo- oophorectomy	M
28	Sopha et al. [45]	1.4 cm, liver	47	RSO for teratoma, SCH + HRT	Right quadrant and back pain	CT		Laparoscopic excision biopsy	S, B
29	Ko et al. [46]	4 cm, right adnexa	64		Recurrent thigh sarcoma	MRI		Laparoscopic BSO	-
30	He et al. [2]	7 × 4.6 cm, left broad ligament	43		Acute lower abdominal pain and hypomenorrhea	USG	pelvic mass torsion	Surgical excision	M
31	Khurana et al. [47]	13 × 9 cm, abdominopelvic	47	Subtotal Hysterectomy for fibroids, bilateral oophorectomy for endometriosis	Vaginal bleeding	CT	leiomyosarcoma	Surgical excision	C, B
32	Tandon et al. [48]	6 × 4.5 cm, liver	50	Laparoscopic hysterectomy with unilateral salpingectomy, endometriosis	Lower abdominal pain	CT	cystic malignancy, metastatic disease or abscess (CT); endometriosis/adenomyosis (liver biopsy)	Surgical excision	M
33	Sampaio et al. [49]	5 cm, abdominal wall	70	Melanoma	Backache	CT	leiomyoma (CT); extrauterine adenomyoma (biopsy)	USG guided core biopsy	M
34	Goswami et al. [50]	20 cm, right broad ligament	46		Swelling and pain abdomen	USG, CT	serous cystadenoma	TAH + BSO	M
35	Paul et al. [1] Case 1	10 cm, pararectal	3	Laparoscopic right ovarian cystectomy 2 years back, endometriosis	Heavy menstrual bleeding, mid-cycle pain and difficulty in initiating micturition	USG		TLH, right oophorectomy, left ovarian cystectomy and excision of pararectal mass	C, B
36	Paul et al. Case 2	3 cm, right round ligament	45	Laparoscopic left ovarian cystectomy 20 years back, SCH 12 yrs back and laparoscopic RSO and left salpingectomy 4 years back, endometriosis	Right lower quadrant pain	USG		Laparoscopic left oophorectomy and excision of the round ligament mass	C, B
37	Paul et al. Case 3	6 cm, pararectal mass; 3 cm, ovarian mass	37	Laparoscopic myomectomy 5 years back, endometriosis	Subfertility, intermenstrual spotting, dysmenorrhea, constipation	USG		Laparoscopic excision and left ovarian cystectomy	C
38	Gruttadauria et al. [51]	3 cm, right ovary; 7 cm, mass anterior to the rectum; multiple masses at the bilateral uterosacral areas and sigmoid mesentery	47	Previous cesarean section	Hip pain	MRI	leiomyomatosis peritoneii or carcinomatosis	Total abdominal hysterectomy, left salpingectomy (right tube was absent), right ovarian cystectomy and excision of multiple masses at the bilateral uterosacral areas and sigmoid mesentery	B
39	Api et al. [52]	52 × 27 mm, left ovary	45	Endometrial polyp	Hypermenorrhea	USG	fibroma or thecoma	Total abdominal hysterectomy and bilateral salpingo-oopherectomy	
40	Belmarez et al. [53]	multiple masses adherent to the rectosigmoid colon, vaginal cuff, and descending colon (from 6 mm to 14.5 cm)	50	Robotic laparoscopic hysterectomy for large uterine fibroids using the morcellation technique, without utilizing a bag	Progressive abdominal bloating and indigestion	USG, CT	malignancy	Bilateral salpingo-oophorectomy, segmental sigmoid colectomy and tumor debulking	B
41	Mandal et al. [54]	90 × 80 × 80 mm, left ovary	60		Pelvic pian	USG	malignancy	Total abdominal hysterectomy and bilateral salpingo-oopherectomy with omental biopsy	M
42	Presented case	63 × 62 × 60 mm, left ovary	40	Endometriosis	Pelvic pain	USG, CT	malignancy	Bilateral salpingo-oophorectomy, omentectomy, peritoneal biopsies	C

Analyzing the past medical history reported, we could classify each case report basing on pathogenesis: Mullerianosis' theory was respected in 52% of cases (22/42), Belmarez's theory (previous gynecological surgery) in 33% of cases (14/42), Cozzutto's theory (coexistence of endometriosis) in 19% of cases (8/42), subcoelomic mesenchymal metaplasia's theory (hormonal treatment response) in 9,5% of cases (4/42) and Rosai's theory (genito-urinary anomalies association) in 2,4 % of cases (1/42).

In nearly one-fifth of cases (8/42), no theory fits with the past medical history and the clinical presentation of each case reported. Due to the lack of data on this rare pathology, no theory is able to globally explain the pathogenesis of extra-uterine adenomyoma so far and more cases collection is needed.

Analyzing the characteristic of extrauterine adenomyoma, abdominopelvic pain is the most common clinical sign at presentation. Endometriosis was reported in the medical history of eleven out of forty-two patients (26%), substantially according to the previous literature review [22] in which endometriotic cyst were identified in the residual ovarian parenchyma of overall 21% of cases. Interesting, slightly more than half of patients (52%) had a previous history of gynaecological surgery for benign pathologies such as hysterectomy, myomectomy or ovarian cystectomy.

Surgical management was the treatment approach in all cases of extra-uterine adenomyomas but only in 2 cases out of 42 a diagnosis of extra-uterine adenomyoma was correctly suspected in the preoperative phase by imaging. Ultrasonography was the most common imaging modality adopted as single diagnostic procedure (66% of the cases, 28/42); more than one radiological staging technique (such as US, CT and MRI) was used in 50% of the cases (21/42).

In sixteen cases out of 42 a preoperative diagnosis was postulated, according to radiological findings or preoperative biopsies: malignancies in 7/16, ovarian thecoma/fibroma in 1/16, ovarian mass torsion in 1/16, myoma in 2/16, endometrioma in 2/16, serous cystadenoma in 1/16, leiomyomatosis peritonei/carcinomatosis in 1/16, and inguinal adenopathy in 1/16. Preoperative biopsies were performed in two cases, reporting extrauterine adenomyoma in one case and a suspect of adenomyosis versus endometriosis in the other one.

Including our presented case, 4 cases (9,7%) were associated with malignancy. Torres et al. [44] reported clear cell adenocarcinoma in a case of broad ligament adenomyoma. Ulm et al. [43] reported focal endometrioid adenocarcinoma in extrauterine adenomyoma (round ligament) with concurrent stage 1 uterine endometrioid adenocarcinoma. Rahilly et al. [23] reported a concurrent occurrence of ovarian adenomyoma with ovarian endometrioid carcinoma and uterine endometrial cancer.

## 4 Conclusion

Extrauterine adenomyoma is still a major challenge. The data available so far bring out the difficulties to correctly diagnose this rare entity preoperatively, due to the lack of a typical ultrasonographic pattern of presentation. This type of ovarian lesion may appear in middle aged women with no previous history of pelvic pain suggestive for endometriosis. The case herein presented shed light on the possibility that ovarian adenomyoma associated with endometriotic cysts may resemble the ultrasound features of ovarian malignancy according to validated IOTA models. The lack of knowledge of this rare entity may eventually lead to unnecessary diagnostic procedures and improper surgical approach.

## Declarations

### Author contribution statement

All authors listed have significantly contributed to the investigation, development and writing of this article.

### Funding statement

This research did not receive any specific grant from funding agencies in the public, commercial or not-for-profit sectors.

### Competing interest statement

The authors declare no conflict of interest.

### Additional information

No additional information is available for this paper.
